# The Ecology of Engagement: Fostering cooperative efforts in health with patients and communities

**DOI:** 10.1111/hex.13571

**Published:** 2022-08-03

**Authors:** Antoine Boivin, Vincent Dumez, Geneviève Castonguay, Alexandre Berkesse

**Affiliations:** ^1^ Canada Research Chair in Partnership with Patients and Communities Montreal Quebec Canada; ^2^ Center of Excellence for Partnership with Patients and the Public, Montreal University Hospital Research Center Université de Montréal Montreal Quebec Canada; ^3^ Department of Family Medicine Université de Montréal Montreal Quebec Canada; ^4^ Faculty of Medicine Université de Montréal Montreal Quebec Canada; ^5^ French School of Public Health (EHESP) Rennes France

**Keywords:** community engagement, ecological models, health coproduction, patient and public involve, patient engagement, theory

## Abstract

**Context:**

Patients and community members are engaged in nearly every aspect of health systems. However, the engagement literature remains siloed and fragmented, which makes it difficult to connect engagement efforts with broader goals of health, equity and sustainability. Integrated and inclusive models of engagement are needed to support further transformative efforts.

**Methods:**

This article describes the Ecology of Engagement, an integrated model of engagement. The model posits that: (1) Health ecosystems include all members of society engaged in health; (2) Engagement is the ‘together’ piece of health and healthcare (e.g., caring for each other, preventing, researching, teaching and building policies together); (3) Health ecosystems and engagement are interdependent from each other, both influencing health, equity, resilience and sustainability.

**Conclusion:**

The Ecology of Engagement offers a common sketch to foster dialogue on engagement across health ecosystems. The model can drive cooperative efforts with patients and communities on health, equity, resilience and sustainability.

**Patients and Public Contribution:**

Three of the authors have lived experiences as patients. One has a socially disclosed identity as a patient partner leader with extensive experience in engagement (individual care, education, research, management and policy). Two authors have significant experience as patients and informal caregivers, which were mobilized in descriptive illustrations. A fourth author has experience as an engaged citizen in health policy debates. All authors have professional lived experience in health (manager, researcher, health professional, consultant and educator). Six patient and caregiver partners with lived experience of engagement (other than the authors) contributed important revisions and intellectual content.

## 1 THE NEED FOR AN ECOLOGICAL PERSPECTIVE ON HEALTH COPRODUCTION

Patients and community members are engaged in nearly every aspect of health ecosystems (e.g., self‐care, clinical care, research, education, public health and policy). Dozens of models and conceptual frameworks have been published to support engagement with patients and community members.^1^, ^2^, ^3^, ^4^

Patient and community engagement tend to assume an intrinsic distinction between care providers (e.g., health professionals) and care receivers (e.g., patients), with patients and citizens being framed as ‘end‐users’, ‘beneficiaries’ and ‘consumers’.^5^ Accordingly, engagement is viewed as ‘inviting people from outside’ into professional health systems.^6^ This assumes that professionals are leading the engagement ‘intervention’ (whom to engage and how) to achieve health systems' goals (e.g., vaccination rates, medication adherence, research uptake and social acceptability of policies).^7^

Increased recognition that health is coproduced with patients and communities calls for more reciprocal and integrated models of engagement, acknowledging that leadership and roles can evolve over time. Coproduction refers to the idea that care is produced jointly by ‘providers’ and ‘users’, thus blurring the distinction between who gives and receives care.^8^ Coproduction of *individual care* recognizes the role of informal care provided by patients, family members and citizens, emphasizing the fact that professional delivery of healthcare services only represents a fraction of the total care received.^9^ From a public health perspective, collective coproduction of *health* recognizes the influence that communities and civic institutions have on environmental and social determinants of health (e.g., mutual support, education, income distribution, employment, climate change).^10^

While the idea of health coproduction has been around for years,^8^ its application in practice remains problematic and inequitable. Engagement practices are still largely characterized by fragmented, short‐term initiatives, leaving important segments of health systems uninviting and not interacting with community members. Also, many communities remain excluded from influential decision‐making roles, thus perpetuating knowledge and power imbalance that impedes global capacities for system transformation and health coproduction.^11^

The International Summit on Patient and Public Partnership—which convened over 100 patient, community and health system leaders—highlighted the need to ‘assemble the puzzle’ and develop a common vision for systemic transformation anchored in a partnership model of health. Participants stressed the need to ‘look at the whole elephant, in interaction with its environment’. International leaders recognized the challenge of building an integrated vision of engagement in health coproduction, emphasizing the need to ‘broaden the tent’ to new ideas and perspectives.^12^, ^13^ Inclusive engagement models are therefore needed to push towards health coproduction for all.^14^

Other disciplines have faced similar challenges in building integrated views of complex systems. In response, ecological models have been fruitfully developed and used in biology, public health, psychology and sociology to understand dynamic and interdependent relationships across systems' levels.^15^ This paper capitalizes on the strengths of ecological models to propose an integrated perspective on engagement in health coproduction.

### 1.1 Objective

The Ecology of Engagement is a conceptual model focused on understanding, supporting and evaluating engagement relationships across health ecosystems. The goal of this article is to describe the Ecology of Engagement and illustrate its potential implications for practice and research. The article is divided into four sections:

1. After a brief description of our theory‐building approach, we sketch an overview of the Ecology of Engagement and offer definitions of key concepts;
2. We then describe the core components of the model: health ecosystems, engagement and the interactions between both;
3. Following the idea that ‘there is nothing more practical than a good theory’,^16^ we illustrate the pragmatic application of the model using international empirical examples;
4. We finally reflect on the strengths and limitations of the model, while outlining a research agenda for the future.

### 1.2 Theory‐building approach

To build the Ecology of Engagement Model, we followed Lynham's interpretive approach to theory‐building in applied research. Theory‐building is understood as ‘the purposeful process or recurring cycle by which coherent descriptions, explanations and representations of observed or experienced phenomena are generated, verified and refined’.^17^ Interpretive theory‐building inquiry focuses on the practical construction of meanings to understand and interpret complex phenomena. Our theory‐building goal was to provide engagement practitioners and scientists with a common language to conceptualize engagement across health systems. As such, we did not seek to inductively build new theories (as in grounded theory approaches) nor did we try to build predictive models based on analytical approaches (as in meta‐analysis). Instead, we examined the fit between existing theories and actual engagement experiences through practice‐to‐theory dialogue based on two sources of expertize: the practical knowledge of patients, professionals and citizens who experienced engagement, and the theoretical expertize of engagement scientists. Our writing team met over a 2‐year period to conceptualize and refine the model and was composed of patients, informal caregivers, citizens, health professionals and researchers with decades of collective engagement experience in different health domains (individual care, education, research, management and policy), disciplines (health services research, medicine, community psychology, management, sociology, anthropology, public health and philosophy) and from different countries (Canada, France, the United Kingdom and the United States). The practical applicability and theoretical coherence of the model were discussed with international experts in engagement science, as well as six patients and caregivers with several years of engagement experience. Our collective approach as a diverse writing team was anchored in dialogical traditions of science highlighting the importance of carefully listening, understanding and learning from multiple paradigms and perspectives while thriving on differences and intellectual tensions.^18^, ^19^ The resulting model should be considered exploratory and descriptive, seeking to pragmatically support further collaboration among practitioners and scientists.^20^

### 1.3 The Ecology of Engagement model overview

The Ecology of Engagement focuses on understanding, supporting and evaluating engagement relationships in health ecosystems. Table 1 offers definitions of key concepts. The core assumptions underpinning the model are:

1. *Health ecosystems include all members of society engaged in health*. Health ecosystems are broader than professional health systems and incorporate patients, informal caregivers communities and their environment as integral contributors to individual and population health.
2. *Engagement is the ‘together’ piece of health and healthcare*. Engagement is dynamic relationships oriented towards joint health‐related activities being carried out together (e.g., caring for each other, doing research together, teaching together, promoting health together and building policies together). Engagement relationships can be categorized according to power and knowledge (activism, information, consultation, participation and partnership), at what levels of health ecosystems they operate (micro‐, meso‐ and macrolevels), among whom (bonding, bridging and linking) and based on its ecosystemic distribution (density, intensity and diversity).
3. *Health ecosystems and engagement are interdependent*. Engagement transforms health ecosystems and is influenced by its ecological context (e.g., social structures, culture, institutions and individuals). Engagement relationships have systemic influences on the equilibrium between health, equity, resilience and sustainability.

**Table 1:** Definitions

Concept	Definition
Health Ecosystems (‘outer core’ of the model)	
Ecology of Engagement	An ecological model focused on understanding, supporting and evaluating engagement relationships at different levels of health ecosystems.
Ecological model	A conceptual framework designed to draw attention to the interdependence between individual and collective determinants of knowledge, attitudes and behaviours. Ecological models assume interaction and multidirectional influence between individuals, collective and environmental factors. Ecosystems are living, open and evolutive systems.
Health Ecosystem	Include all members of society engaged in health‐related activities. Health‐related activities include caring for self or others, health promotion, research, education, governance and policy. Health ecosystems are broader than professional health systems and incorporate patients, informal caregivers, communities and their environment as integral contributors to individual and population health.
Community system	Community members (patients, family members, informal caregivers and citizens) and community groups (patient associations, networks, civic institutions and social movements) who engage in health‐related activities.
Professional system	Professional members (e.g., clinicians, managers, researchers, policymakers) and institutions (healthcare institutions, public health agencies, government) who engage in health‐related activities.
Microlevel	Individual‐level relationships (e.g., individual patient and professionals' decision‐making).
Mesolevel	Groups and institutions in which individuals interact together (e.g., patient association, clinic, research team, training institutions).
Macrolevel	Forces within the larger social system in which groups and institutions are embedded (e.g., culture, policies, values, legislation, environment).
Engagement relationships (‘inner dynamic’ of the model)	
Engagement	Dynamic relationships among individuals or groups oriented toward joint health‐related activities. Engagement is the ‘together’ piece of health and healthcare (e.g., caring together, doing research together, teaching together, promoting health together and developing policies together).
Typology of engagement	Characterization of engagement relationships according to power and knowledge flow (activism, information, consultation, participation and partnership), at what levels of health ecosystems they operate (micro‐, meso‐ and macrolevels), and among which individuals and groups (e.g., bonding, bridging and linking).
Information	Engagement relationship where knowledge is communicated from engagement leaders to engaged individuals or groups.
Consultation	Engagement relationship where knowledge is collected from engaged individuals/groups toward engagement leaders.
Participation	Engagement relationship where knowledge is exchanged between engagement leaders and engaged people.
Partnership	Engagement relationship where engaged parties colead (governance), cobuild (process) and are coaccountable (results) for a common initiative being carried together.
Activism	Engagement relationship where engagement leaders challenge existing power relationships and social structures to change the status quo (including social norms, embedded practices, policies or the dominance of certain social groups).
Bonding	Engagement relationships within community or professional systems. Bonding relationships are ‘inward looking’, reinforcing connections among homogenous groups of people with a shared identity (e.g., self‐support group, professional team)
Bridging	Engagement relationships across community or professional systems. Bridging relationships are ‘outward looking’, reinforcing connections across a heterogeneous group of people with different identities (e.g., quality improvement committees with patients and health professional members).
Linking	Engagement relationships across ecosystem levels. Linking relationships are ‘upward looking’, reinforcing connections with people across power and authority gradients (e.g., tenants' participation in a social housing management committee, project coleadership between a patient and health authority manager, community development project with citizens and municipal government leaders).
Density	Total number of engagement relationships among individuals and groups. Density quantifies how many engagement relationships are developed and maintained among community members and professionals, each offering opportunities for mutual influence across the whole ecosystem.
Intensity	Strength of bonding, bridging and linking engagement relationships within the health ecosystem as a whole, allowing sustained connections within and across community and professional systems.
Diversity	Variety of individuals and groups forging engagement relationships within the health ecosystem as a whole. Diversity points to the inclusiveness of engagement relationships, and the potential exclusion of certain individuals and groups with whom knowledge, power and health production capacity may be unequally distributed.
Ecological effects of engagement (‘inputs and outputs’ of the model)	
Health coproduction	Change in the health of individuals and groups resulting from the joint actions of community members and professionals.
Resilience	Ability of health ecosystems to adapt, evolve and survive as a result of changes and crises.
Resource use	Use of human, financial or environmental resources by the health ecosystem.
Equity	Inclusive distribution of knowledge, power and health production capacity among individuals and groups within health ecosystems.

## 2 HEALTH ECOSYSTEMS INCLUDE ALL MEMBERS OF SOCIETY ENGAGED IN HEALTH

Ecological perspectives bring to the foreground a number of key assumptions in the understanding of health systems and their relationships to engagement.

First, *‘health systems’ are viewed as ‘ecosystems’* that are not restricted to professional services and institutions (e.g., health professionals, hospitals and clinics, research institutions and health ministries). Health ecosystems incorporate patients, informal caregivers, citizens, communities and their environment as integral components of health production.^8^

Ecological systems are ‘*living systems*’. Mechanical systems (e.g., cars, the solar system) operate under constant, predictable rules. Conversely, living systems (e.g., the human body, a forest) are characterized by complexity and emergent properties arising across levels.^21^ Individuals are embedded within communities, institutions and societies with emergent properties across levels^22^ (e.g., collective ability to shape policy decisions). Engagement among individuals is qualitatively different from collective engagement across communities, institutions and groups, which have emergent properties beyond those of its participating members.

Ecological perspectives emphasize the *interdependence and multidirectional influence between individual and collective factors*.^15^ Central to ecological perspectives are the assumption of mutual interaction and reciprocal causation among levels. The microlevel refers to individuals (e.g., individual patient and professional decision‐making). The mesolevel refers to groups and institutions (e.g., patient association, clinical team, community support group and research institution). The macrolevel refers to larger forces within the social systems in which groups and institutions are embedded (e.g., environment, culture, policies and legislation). From an engagement perspective, engagement at the microlevel (e.g., care) can generate knowledge and opportunities for engagement at the meso‐ (e.g., institution) and macrolevels (e.g., policy). Conversely, engaging patients and professionals at the meso‐and macrolevel (e.g., change in legislation) can transform the opportunities for engagement at the individual level.

Ecological systems are ‘*open systems*’, drawing energy from the outside environment. Health systems cannot be isolated from broader economic, societal and environmental systems. Living systems like the human body or the health system need to constantly extract energy from their environment to survive.^23^

Ecological systems are *evolutive*: individual trees are born, grow and die, just like individuals evolve in their health status, knowledge, care capacity and relationships. Instability is an intrinsic feature of ecological models, which can be influenced by internal changes (e.g., pandemics) or external changes (e.g., climate change).^24^ Health ecosystems are transformed by historical changes in culture, technologies, economic growth, social inequalities, population change and crisis. Resilience refers to the ability of individuals and systems to adapt, evolve and survive as a result of change and crisis.

In sum, the Ecology of Engagement proposes an ecological perspective on health systems, which are reconceptualized as ‘health ecosystems’ that include all members of society potentially engaged in health coproduction. Health ecosystems are open, living and evolutive systems characterized by interdependent relationships across individuals and groups.

## 3 ENGAGEMENT IS THE ‘TOGETHER’ PIECE OF HEALTH AND HEALTHCARE

### 3.1 Defining engagement

Dozens of definitions of engagement have been proposed in the literature.^25^ Grounded in an ecological perspective, our definition of engagement is relationship‐focused and systemic. We define engagement as dynamic relationships oriented towards joint health‐related actions. We assume that engagement represents a specific type of relationship between individuals and groups, focused on actions being carried together. This broad definition of engagement does not mean that ‘everything in health is engagement’ but that ‘engagement can be related to any component of health ecosystems’. Engagement is the ‘together’ piece of health and healthcare: caring together, doing research together, teaching together, designing innovations together, promoting health together, defining community priorities together, educating children together and building policies together.

To unpack this umbrella definition, we propose a typology of engagement relationships according to power and knowledge flow (activism, information, consultation, participation, partnership), at what levels of health ecosystems they operate (micro‐, meso‐ and macrolevels), among which individuals and groups (e.g., bonding, bridging, linking), and their ecological distribution (density, intensity and diversity).

### 3.2 ‘The Loop’: Distinguishing engagement based on knowledge and power

Following other authors,^2^ we assume that theoretically relevant differences exist between engagement relationship categories.^1^, ^2^, ^3^ A common assumption of existing typologies is that engagement is either led by professionals (e.g., Rowe and Frewer ‘typology of public engagement mechanisms’ ranging from ‘information’ to ‘participation’^2^) or led by citizens themselves (e.g., Sherry Arstein's ‘ladder of citizen participation’ ranging from ‘manipulation’ to ‘citizen control’^1^).

**Figure 1:**
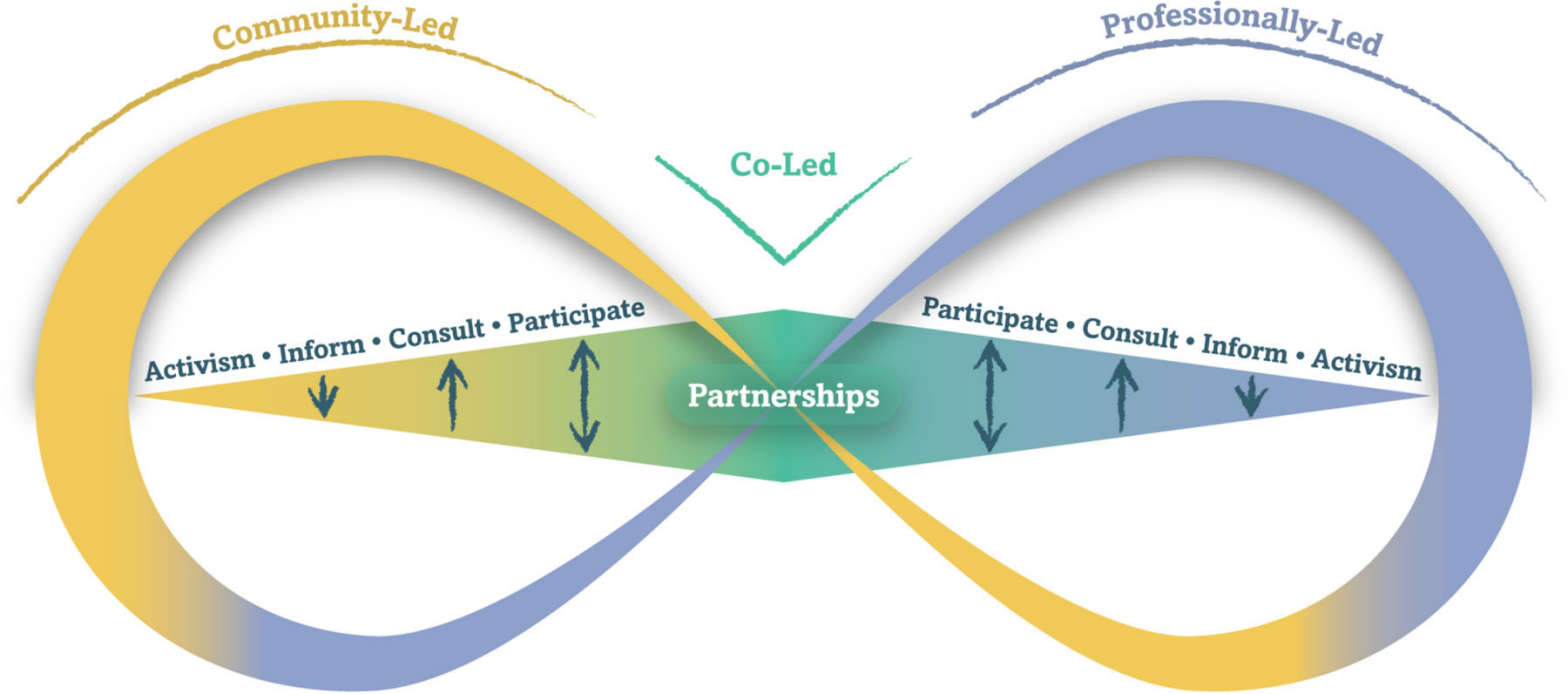
The loop (engaging how and under whose leadership). Engagement are dynamic relationships oriented toward joint health‐related actions. Engagement can be led by the community (yellow), by professionals (blue), or co‐led (green). Engagement is distinguished by knowledge flow (information *to*, consultation *from*, participation *between*) and power (activism to transform power dynamics from the *outside*, partnering by sharing power from the *inside*). Engagement relationships evolve over time (‘bouncing along the loop’).

The Ecology of Engagement adopts a reciprocal perspective on engagement: the ‘*Engagement Loop*’ (Figure 1). A loop is a symmetrical figure representing a process the end of which is connected to the beginning. The Engagement Loop is:

1. *Reciprocal*, recognizing that engagement relationships can be led by professionals, community members or both. Unlike a scale or ladder, the loop does not have a top or a bottom (specifying ‘higher’ or ‘lower’ levels of engagement), but two poles of attraction at each end (community vs. professionally‐led engagement) that can be more or less powerful or balanced at different points in time;
2. *Dynamic*, highlighting the shifting nature of engagement relationships over time. For example, a patient can take leadership of self‐care activities at home, be led by professionals during hospitalizations and engage in partnership decision‐making during outpatient follow‐up appointments. This shift in engagement leadership can also be observed at the collective level, where professionally led initiatives can transition to community‐led programmes over time, and vice‐versa.^26^

The Engagement Loop distinguishes between five types of engagement relationships according to power (who frames engagement goals and approach) and knowledge (in which direction knowledge is produced and exchanged between individuals and groups).^1^, ^2^, ^3^

Information, consultation and participation approaches are distinguished by the direction of knowledge flow. Knowledge is either communicated *to* (*information*), collected *from* (*consultation*) or exchanged *between* individuals and groups (*participation*).^2^ Information, consultation and participation assume unilateral leadership of the engagement process by professionals or community members. Reciprocity of the typology allows consideration of situations where community members are acting as engagement leaders (e.g., community‐led information campaign targeting professionals) or the other way around (e.g., professionally‐led education programme).

Partnership and activism approaches are distinguished by power relationships. *Partnership* is characterized by power sharing and coleadership. Relationship‐building and power‐sharing are assumed as preconditions of engagement.^27^ Conversely, *activism* targets the transformation of power relationships and social structures as a potential result of engagement.^28^, ^29^ Partnership approaches seek to achieve ‘transformation from the inside’ (through collaborative leadership, sharing of expertize and resources and partnership synergy),^27^ while activism focuses on changing power relationships and institutional structures ‘from the outside’ (through critical questioning of discourses and redistribution of resources and power).^30^, ^31^

Differences in engagement approaches are reflected in the choice of appropriate evaluation criteria to assess the process and outcomes of engagement. Table 2 illustrates examples of engagement at different health ecosystem levels, along with their potential evaluation criteria.

**Table 2:** Examples of engagement relationships and evaluation criteria

Engagement category	Definition	Engagement methods	Microlevel engagement examples	Meso/macrolevel engagement examples	Evaluation criteria
Information	Knowledge is communicated *from* engagement leaders *to* engaged individuals/groups	Education material Decision aids Online information Conference Webinar Media	Professional‐led: Professional informs the patient on individual long‐term risks of cardiovascular disease.	Professional‐led: Professional education programme to raise public awareness of mental health issues.	Process: Information content, quality, understanding and reach. Outcomes: Change in awareness, knowledge, attitude and behaviours of people engaged.
			Community‐led: Patient takes the initiative of informing professional on side effects of medication.	Community‐led: Patient‐led education programme where patients train health professionals on relationship skills.	
Consultation	Knowledge is collected *from* engaged individuals/groups *towards* engagement leaders	Interview Survey Focus group Delphi Advisory committee	Professional‐led: Professional consults patient on his preferences for home or institution‐based palliative care, but the orientation of care is ultimately based on a clinical algorithm.	Professional‐led: Research team set up a patient‐advisory committee for input into research questions or guideline recommendation.	Process: Representativeness, reliability, generalizability. Outcomes: Change in knowledge, attitudes and behaviours of engagement leaders, influence on policies.
			Community‐led: Patient consults professional for advice on the use of over‐the‐counter vitamin supplements and makes the final decision by him/herself.	Community‐led: Community group consults scientific advisors to validate the content of public campaign documents	
Participation	Knowledge is *exchanged between* engagement leaders and engaged people.	Committee participation Deliberative meeting Consensus conference Nominal group technique Deliberative polling Task force Town hall meeting	Professional‐led: Parents contribute to implementing a professionally led home‐care plan for a sick child by exchanging information with the clinic.	Professional‐led: Professionally led practice guideline group invite patients to the guideline development panel.	Process: Information exchange, inclusivity, independence, fairness. Outcome: Mutual learning and influence between engaged parties, informed collective proposal, influence on policies.
			Community‐led: Parents initiate deliberation with clinical ethicists on care options for their child while keeping leadership on the decision.	Community‐led: Parent association host deliberative panel between clinicians, researchers, youths and parents to improve mental health services.	
Partnership	Engaged parties colead (governance), cobuild (process) and are coaccountable (results) for a common initiative being carried together.	Codesign Shared decision‐making^a^ Coconstruction^a^ Coproduction^a^	Co‐led: Shared decision‐making between patient and professional in establishing clinical agenda, generating and implementing care options, coassessing progress and being coresponsible for health results.	Co‐led: Community members and professionals colead a project where patients, clinicians and citizens care together for people with social and medical issues.	Process: Partners' relationships; sharing of power, resources and information; trust. Outcome: Partnership synergy, sustained collaborative efforts, collective production of care, knowledge, services and policies.
Activism	Engagement leaders challenge existing power relationships and social structures to change the status quo (including social norms, embedded practices, policies or the dominance of certain social groups).	Protest Lawsuit (Social) media campaign Civil disobedience Petitions Boycott Walkouts	Professional‐led: Medical activism to challenge health insurer coverage restriction for a single patient.	Professional‐led: Health professionals initiate media campaigns to change tobacco policies.	Process: Coalition‐building, prepare/communicate convincing data and rationales, lobby policymakers. Outcome: Change in power distribution, group dominance, institutional structure, social norms, practices and policies.
			Community‐led: Patient advocates for his/her right to access a specific treatment from his/her insurer.	Community‐led: Women groups challenge medical dominance over childbirth and advocate for legal recognition of midwives.	

^a^ Engagement approaches labelled as ‘codesign’, ‘shared decision‐making’, ‘coconstruction’ or ‘coproduction’ are best classified in the partnership category when leadership is shared between parties. However, they are best classified in the information, consultation or participation category when leadership is held by a single party (e.g., ‘codesign’ of health technology led by engineers where patients are consulted at the initial or pilot testing stage, ‘coproduction’ of research led by scientists where citizens are engaged in crowdsourcing of data collection, ‘shared decision‐making’ on antidepressant medication choice where agenda‐setting is led by clinicians only and patient is consulted on the choice of drug).

^b^Engagement approaches labelled as ‘committee participation’ are best classified in the consultation category when the committee is structured around a collection of opinions, feedback, advice and recommendations with little or no information exchange (membership in a citizen advisory council). The defining characteristic of participation methods is two‐way interaction and influence between engagement leaders and participants.

### 3.3 Engagement with whom: Bonding, bridging and linking

**Figure 2:**
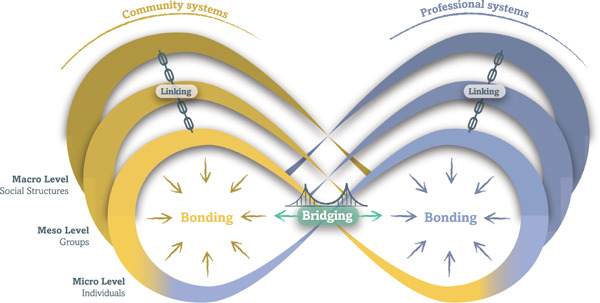
Engagement relationships can be distinguished according to who is engaged and at what level. Bonding are ‘inward looking’ engagement relationships among community members (yellow) or professionals (blue). Bridging are ‘looking across’ engagement relationships *between* community and professional groups (green). Linking are ‘upward looking’ engagement relationships across power authority and levels (black).

Drawing from social capital theory, we further distinguish engagement relationships according to who is engaged with whom (Figure 2)^32^, ^33^:

1. *Bonding* refers to engagement relationships *within* community or professional systems. Bonding relationships are ‘inward looking’, reinforcing connections among homogenous groups of people with a shared identity (e.g., a patient association, peer support group, research network or professional team).
2. *Bridging* refers to engagement relationships *across* community or professional systems. Bridging relationships are ‘outward looking’, reinforcing connections across a heterogeneous group of people with different identities (e.g., care team with peer‐support workers and health professionals working together; governance committees with patients and managers; research teams with academic researchers and citizens).
3. *Linking* is ‘upward looking’, reinforcing connections with people across power, authority gradients and levels (e.g., tenants' participation in a social housing management committee, project coleadership between a patient and health authority manager, community development project with citizens and municipal government leaders).

Bonding and bridging relationships are distinguished by an open and fluid distinction between community and professional identities based on socially constructed identities, rather than static and predetermined characteristics.^34^, ^35^ For example, the same individual may identify and be socially labelled as ‘patient’, ‘physician’, ‘researcher’ or ‘citizen’ depending on their own self‐identification and roles. Distinguishing between community and professional identities remains conceptually and pragmatically useful to understand engagement relationships within health ecosystems. Holding a socially recognized identity of ‘physician’ opens certain bonding and linking relationship opportunities (e.g., professional colleague or manager) that are different if the same individual self identifies as a ‘patient partner’.

### 3.4 Ecosystemic distribution of engagement relationships

Engagement relationships can be studied ‘from the inside out’, by characterizing the relationships of a focal individual or collective (e.g., bonding and bridging relationships established by a single patient partner) or studied from the ‘outside‐in’, by characterizing the distribution of engagement relationships within a health ecosystem.^36^ The ecosystemic distribution of engagement relationships can be characterized based on its density, intensity and diversity:

1. *Density* refers to the total number of engagement relationships among individuals and groups within an ecosystem. It quantifies how many engagement relationships are developed and maintained, thus offering opportunities for influence across the whole ecosystem.
2. *Intensity* refers to the strength of engagement relationships within the health ecosystem. Stronger engagement relationships (e.g., team membership) tend to allow for more constant and sustained connections within and across community and professional systems, while weaker ties (e.g., informal contacts among individuals) can play a role in bridging more isolated community and professional groups.^37^
3. *Diversity* refers to the variety of individuals and groups forging engagement relationships within the health ecosystem as a whole. Diversity points to the inclusiveness of engagement relationships and the potential exclusion of certain individuals and groups with whom knowledge, power and health production capacity may be unequally distributed.

## 4 HEALTH ECOSYSTEMS AND ENGAGEMENT ARE INTERDEPENDENT

**Figure 3:**
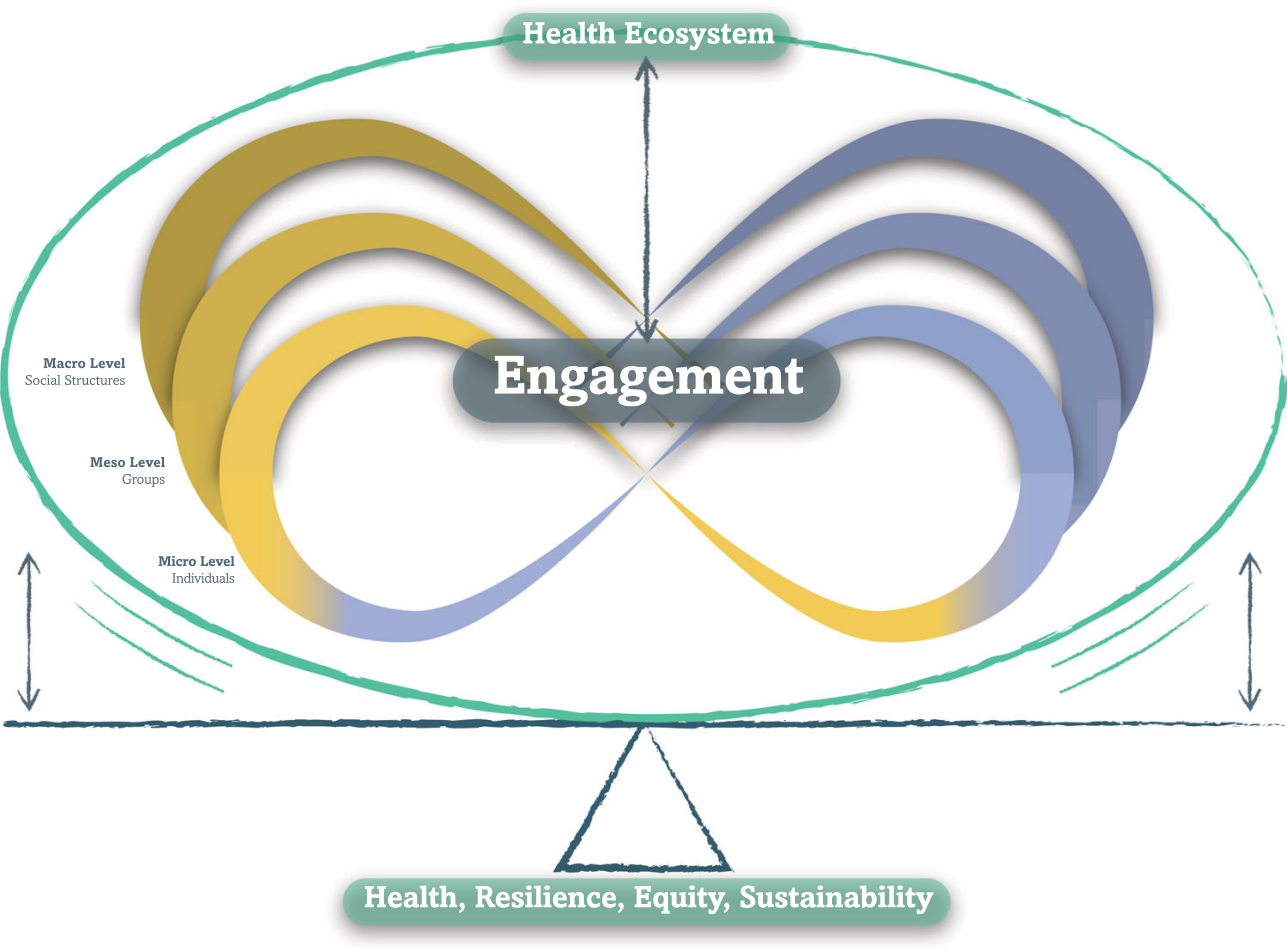
Health Ecosystems and Engagement are interdependent. Engagement transforms Health Ecosystems and is influenced by it. Engagement relationships have systemic effects on the equilibrium between health, resilience, equity and sustainability.

The Ecology of Engagement assumes a bidirectional influence between engagement and health ecosystems: engagement transforms ecosystems and is shaped by its ecological context. Engagement relationships have systemic effects on the equilibrium between health, resilience, equity and sustainability (Figure 3). This section illustrates the interdependence between engagement and health ecosystems.

### 4.1 Engagement transforms health ecosystems

A growing empirical literature has documented how the engagement of patients, professionals and communities can transform its ecological context by shaping policies, social determinants of health, research, health institutions, education programmes and direct care.^38^, ^39^, ^40^, ^41^, ^42^, ^43^, ^44^ Similarly, several ecological factors (e.g., culture, institutions, policies) influence the process and outcomes of engagement.^45^, ^46^, ^47^, ^48^ These influences can operate ‘upstream’ (micro‐ to macrolevels), ‘downstream’ (macro‐ to microlevels) or ‘laterally’ (across a single level).

The example of patient engagement in medical education helps illustrate this transformative impact of engagement on health ecosystems. A number of medical schools are engaging with patients as teachers.^49^, ^50^ The decade‐long experience of patient engagement in medical education at Université de Montreal (Canada) illustrates how long‐term engagement in education can have ecological effects on research, policy and individual care. Initiated in 2010 under the coleadership of the faculty of medicine dean and a patient leader, the ‘Montreal Model’ has grown into one of the largest patient partnership training programmes internationally (over 250 paid patient educators training 1500 health professionals from 14 health disciplines every year).^50^ Over time, patient engagement in professional education influenced the (lateral) engagement of patients in health governance institutions, which created momentum for (upstream) policy reforms on engagement in quality improvement, research and accreditation.^51^ Patient engagement in education also had (downstream) influence on individual care through its kindling effects on peer support in clinical care and national training programmes for informal caregivers.^52^ Overall, this experience illustrates the potential long‐term relationships between engagement in education, policy, research, care and health and its ecological impacts across health ecosystems.

### 4.2 Engaging communities and professionals in health coproduction

Health ecosystems can move back and forth between community‐led, professionally led or co‐led health production systems as a result of historical, technological, political and cultural forces. For example, low‐income countries tend to rely heavily on community health production systems (e.g., informal care by family and community members). Community structures tend to be marginalized with technological progress and the professionalization of care.^48^, ^52^ Conversely, extreme professionalization of care tends to be limited by its high resource use and inability to reach certain underserved communities, which creates counter‐balancing forces towards coproduction models built on the synergy between community and professional resources.

Bridging engagement relationships between professionals and community members can also support health coproduction dynamics.^26^ For example, peer‐support workers foster health coproduction through their ability to build trustful relationships across professionals and community systems. This bridge can operate from the clinic to the community (e.g., peer‐mentors connecting chronic disease patients living in poverty with community organizations offering housing and food support),^52^ or from the community to the clinic (e.g., peer‐support workers helping to reconnect people experiencing homelessness with professional healthcare services or harm‐reduction strategies aligned with people's own goal and values).^49^ Community members can also foster bonding (e.g., introduction to mutual support group) and linking relationships (e.g., advocating for service delivery adaptation).

### 4.3 Engagement influences resilience, equity and sustainability

Engagement relationships have potential effects on health ecosystems' resource use (entropy), adaptation to change and crisis (resilience) and disparities (equity).

An ecological perspective suggests that the diversity and intensity of engagement relationships influence health ecosystems' *sustainability* (use of human, financial and environmental resources).^50^ Several initiatives mobilizing patients, citizens and communities as care partners have shown comparable or improved outcomes compared to professionally led models of care,^51^, ^53^ with lower use of resource‐intensive services (e.g., emergency room visits and hospitalizations) and overall costs of care.^54^, ^55^, ^56^

The environmental impacts of different health production systems further illustrate the relationships between engagement and sustainability. Climate change has significant impacts on global health and the healthcare sector is a direct contributor to climate change.^57^, ^58^ Professional health production systems tend to produce higher (environmental) effects than community‐based or health coproduction systems. Large urban hospitals tend to have a high carbon footprint because of direct energy use, disposable technologies and transportation.^58^ Conversely, community‐based care focused on people empowerment and self‐management has greater potential for low carbon footprint healthcare.^57^ Engagement relationships can also influence the entropic effects of health on the environment, through professional and citizen mobilization efforts.^59^, ^60^

*Equity* is also influenced by engagement. Despite its massive investment in healthcare (17.9% of gross domestic product), the United States ranks last among developed countries for many health indicators notably because of persistent inequalities according to socioeconomic status, race and gender^14^, ^61^ Although the density and intensity of patient and family engagement programmes is relatively well developed in the United States (e.g., shared decision‐making, public performance reports of health coverage plans, self‐management programmes), the engagement of a diversity of groups remains problematic, particularly with disadvantaged and marginalized communities.^14^ Local engagement strategies initiated by or developed with marginalized groups have shown promising results in reducing health inequities, especially when they can address structural and power issues.^53^, ^62^ The effects of engagement on equity can either increase or decrease health inequalities, depending on the diversity (and power) of engagement relationships.

Finally, engagement relationships can affect *resilience*, defined as the ability to adapt, evolve and survive as a result of changes and crises.^32^ High degrees of interactions among individuals and groups act as stabilizing forces to counter the effects of ecological instabilities.^24^ Ecological systems with more dense, diverse and intense relationships (e.g., forests) are more likely to adapt to environmental changes than homogenous ecosystems characterized by low diversity and interactions (e.g., agricultural monoculture). Within the health context, empirical studies have highlighted the contribution of engagement relationships to resilience, as exemplified by the Ebola and COVID‐19 pandemics.^32^

The 2014–2015 Ebola outbreak in West Africa had mortality rates of approximately 70% and occurred in the context of fragile and under‐resourced professional healthcare systems. Community engagement thus became a pillar of intervention to reduce the progression of the disease, including the design and planning of public health response with communities; building public trust, communication strategies; and community engagement in case detection, follow‐up and quarantine. These measures directly contributed to controlling the outbreak and supporting the resilience of the health ecosystem in a concerted effort with its limited professional structure.^63^, ^64^, ^65^ The response to COVID‐19 offers a contrasting perspective on resilience in professionally led health ecosystems. Equipped with well‐resourced healthcare systems, countries in North America and Europe have heavily relied on professional strategies and centralized decision‐making. Governance of national COVID‐19 strategies has been dominated by professional experts, with little direct input from patients and civil society.^66^, ^67^ Community groups have most often been mobilized as delivery mechanisms, with little decision‐making power over the design and planning of local pandemic response.^68^ While the importance of community engagement has been recognized in later phases of the pandemic,^68^, ^69^, ^70^ over‐reliance on professional care during the first wave of COVID resulted in rapid overloads of professional health systems.^71^, ^72^, ^73^

In summary, the Ecology of Engagement points towards systemic impacts of engagement, through its transforming influence on individuals, institutions and policies, as well as its ecological effects on health, resilience, equity and sustainability.

## 5 STRENGTHS, LIMITATIONS AND AREAS FOR FUTURE DEVELOPMENT

The main contribution of the Ecology of Engagement is to offer an integrated perspective on engagement and health. It proposes a conceptually coherent model helping to bridge engagement practice and science. Because social interactions are embedded in nearly all health activities, the Ecology of Engagement offers a useful theoretical architecture to connect engagement relationships across domains (e.g., care, research, education, health promotion and policymaking) and levels (micro‐, meso‐ and macrolevels).^32^, ^36^, ^74^ The proposed typology of engagement offers a reciprocal perspective on engagement leadership by community members and professionals. The use of a descriptive typology also helps disentangle engagement approaches labelled with generic terms hiding important differences in theory and practice.^75^ Finally, the model proposes core assumptions about the potential systemic effects of engagement on health coproduction, resilience, equity and entropy. The model is applicable to concrete examples and can drive further action and research.

We present the Ecology of Engagement model with humility, acknowledging that the model is building on the work of many others. The integrative nature of the Ecology of Engagement seeks to connect engagement theories that are often disconnected from each other while offering a common language to support further dialogue. This does not imply that the model offers a comprehensive theory of engagement in health. It is merely offered as a pragmatic sketch to build upon.

Drawing from multiple scientific and epistemic traditions can create paradoxes that are difficult to reconcile (e.g., postpositivist views of entropy coexisting alongside constructivist views of social identities), and is influenced by the perspectives of experts involved in theory‐building.^76^ Such internal tensions are intrinsic to transdisciplinary undertaking and should inform further research and practice dialogue.

The idea that patients, professionals and communities can be engaged at the micro‐, meso‐ and macrolevels has been proposed elsewhere^34^ and our main contribution is to add a common theoretical architecture to connect engagement among domains and levels. The model bridges the engagement literature with broader research traditions on ecological approaches to health and social capital.^15^, ^32^, ^36^, ^74^ Proposed categories of the engagement typology (e.g., information, consultation, participation, partnership and activism) are not new^1^, ^2^ and the main innovation is to present them in a reciprocal perspective to describe engagement relationships led by professionals, communities or both. The evolutive nature of engagement presented in this paper resonates with other scholars looking at engagement as a ‘journey’ that transforms its actors and context over time.^77^

Theoretical models perform a central role in mediating between science and practice.^78^ Because of its broad perspective, translation of the Ecology of Engagement into a specific engagement domain and projects will require adaptation. For example, an ecological perspective of engagement in research requires clarification of relevant institutions (e.g., research centres), policies (e.g., research policies), social norms (e.g., what is recognized as valid knowledge) and individual factors (e.g., researchers' attitude toward engagement) that can affect engagement.

*Future research*: As simplified versions of complex realities, ‘all models are wrong but some are useful’.^79^ Readers should keep in mind that the Ecology of Engagement presents a set of organized hypotheses to be tested by empirical research (e.g., the density, diversity and intensity of engagement relationships influences health coproduction, resilience, equity and entropy). ‘A theory is a tender growth, naturally imperfect when first proposed’ and requires further testing, refinement and critical questioning.^16^ Ecological perspectives, because of their multilayered and dynamic nature, should raise caution about simple statements of cause and effects (e.g., ‘more engagement leads to better health at a reduced cost’). Further research, critical testing and refinement of the model are required:

1. First, there is a need to synthesize what is known about ecological factors influencing engagement. A number of reviews have highlighted social, organizational and individual‐level factors that influence the engagement process and outcomes.^47^, ^48^ Conversely, abundant literature has documented the impacts of engagement on individuals, institutions and policies.^18^, ^38^, ^39^, ^40^, ^41^, ^42^, ^43^, ^44^ However, this literature remains highly fragmented and siloed (e.g., engagement in care, education, research, quality improvement, service delivery, health promotion and policy). The literature is in need of an ‘ecological synthesis’ to reconnect the pieces together and build a more comprehensive view of the ecosystemic relationships between engagement and health. Such an integrated approach to research is pragmatically important to build coherent engagement policies across sectors, as opposed to fragmented and short‐lived engagement support strategies.
2. The Ecology of Engagement Model reframes engagement as dynamic relationships embedded in evolutive ecological systems, rather than framing engagement as a static ‘intervention’ or ‘method’ designed by professionals. An instrumental approach to engagement is prominent in the evidence‐based medicine literature, which has sought to test the effectiveness of engagement in achieving predefined outcomes.^19^ Adoption of an ecological perspective pushes researchers to approach engagement differently, calling for explicit consideration of the mutual influences between engagement and its ecological context. It also points towards the unexpected effects of engagement beyond its predefined objectives, as professionals and community actors are transformed by their engagement relationships. It also underlines the potential for engagement to reshape the ecological context in which it is embedded by transforming organizations, communities, culture and social norms. This constant interplay between engagement and its ecological contexts requires innovative, flexible and dynamic approaches to research and evaluation.^80^, ^81^
3. Finally, the primary literature is in need of theoretically sound research on the long‐term influence of engagement over time (e.g., how mesolevel engagement of patients in research can have ‘upstream’ influence on macrolevel policies or ‘downstream’ influence on individual care and population health). An ecological perspective calls for long‐term follow‐up of the rippling effects of engagement, an important gap in the existing scientific literature that is still dominated by short‐term descriptive studies of engagement initiatives.^20^

## 6 CONCLUSION

In conclusion, the Ecology of Engagement opens new avenues for engagement practice and science by proposing an integrated, interdependent and reciprocal model of engagement in health ecosystems. Grounded in a coproduction of health perspective, the model adopts a reciprocal typology of engagement relationships among individuals and groups, acknowledging dynamic changes in the leadership between community members and professionals. The model can serve as a common sketch to foster dialogue between system leaders, engagement practitioners and scientists to drive cooperative efforts on health system improvement, sustainability, resilience and equity. Future research should strengthen ecological perspectives on engagement, drawing from decades of experience in other research disciplines.

## AUTHOR CONTRIBUTIONS

Antoine Boivin was involved in the conceptualization of the Ecology of Engagement Model and typology, drafting the manuscript and visual representation. Vincent Dumez, Geneviève Castonguay and Alexandre Berkesse contributed important intellectual input to the model conceptualization, description, visual representation and illustrative examples. All reviewed and approved the final version of the manuscript.

## CONFLICT OF INTEREST

The authors declare no conflict of interest.

## Data Availability

The data availability statement does not apply.
