# Correction to: The ecology of engagement: Fostering cooperative efforts in health with patients and communities

**DOI:** 10.1111/hex.13911

**Published:** 2024-04-13

Some discrepancies were identified between citation numbers and bibliographic references, after publication of the article:

A. In paragraph 1 of **1.2 Theory‐building approach**, ‘Our collective approach as a diverse writing team was anchored in dialogical traditions of science highlighting the importance of carefully listening, understanding and learning from multiple paradigms and perspectives while thriving on differences and intellectual tensions’.^18,19^

- Reference #18 should refer to: Johnson RB. Dialectical pluralism. *J Mix Method Res*. 2017;11(2):156–173.
- Reference #19 should refer to: Guba EG, Lincoln YS. Paradigmatic controversies, contradictions, and emerging confluences. In: *The Sage Handbook of Qualitative Research*, 3rd ed. Sage Publications Ltd; 2005:191–215.

C. In paragraph 1 of **1.2 Theory‐building approach**, ‘The resulting model should be considered exploratory and descriptive, seeking to pragmatically support further collaboration among practitioners and scientists’.^20^

- Reference #20 should refer to: Pluye P, El Sherif R, Hong QN, Vedel I. The pluralism of scientific worldviews in mixed methods research: Where do I stand?

D. In paragraph 2 of **4.1 Engagement transforms health ecosystems**, ‘A number of medical schools are engaging with patients as teachers’.^49,50^

- Reference #49 should point toward: Morris G, Simon G, Debra T, Michael R, Ernie M, Angela M. Patient/service user involvement in medical education: a best evidence medical education (BEME) systematic review: BEME Guide No. 58. Med Teach. 2019;1–13.
- Reference #50 should refer to: Karazivan P, Dumez V, Flora L, Pomey MP, Grande CD, Ghadiri DP, et al. The patient‐as‐partner approach in health care. Acad Med. 2015;90(4):1.

F. In paragraph 2 of **4.1 Engagement transforms health ecosystems**, ‘Over time, patient engagement in professional education influenced the (lateral) engagement of patients in health governance institutions, which created momentum for (upstream) policy reforms on engagement in quality improvement, research and accreditation’.^51^

- Reference #51 should refer to: Boivin A, Flora L, Dumez V, l'Espérance A, Berkesse A, Gauvin F. Co‐construire la santé en partenariat avec les patients et le public: historique, approche et impacts du «modèle de Montréal». In: C Hervé JS, eds. *La participation des patients, Ethique biomédicale et normes juridiques*. Dalloz; 2017.

G. In paragraph 2 of **4.3 Engagement influences resilience, equity and sustainability**, ‘An ecological perspective suggests that the diversity and intensity of engagement relationships influence health ecosystems' sustainability (use of human, financial and environmental resources)’.^50^

- Reference #50 should refer to: Chapin FS, Torn MS, Tateno M. Principles of ecosystem sustainability. Am Nat. 1996;148(6):1016–1037.

H. In paragraph 4 of **5 Strengths, limitations and areas for future development**, ‘The idea that patients, professionals and communities can be engaged at the micro‐, meso‐ and macrolevels has been proposed elsewhere’^34^ […].

- Reference #34 should refer to: Carman KL, Dardess P, Maurer M, Sofaer S, Adams K, Bechtel C, et al. Patient and family engagement: a framework for understanding the elements and developing interventions and policies. Health Affair. 2013;32(2):223–231.

We apologize for these errors.

